# Assessing Epstein–Barr virus in gastric cancer: clinicopathological features and prognostic implications

**DOI:** 10.1186/s13027-023-00489-9

**Published:** 2023-02-19

**Authors:** Guanghua Li, Zhihao Zhou, Zhixiong Wang, Zhao Wang

**Affiliations:** Department of Gastrointestinal Surgery, First Affiliated Hospital of Sun Yat-Sen University, Zhongshan 2nd Street, No. 58, Guangzhou, 510080 Guangdong China

**Keywords:** Gastric cancer, Epstein–Barr virus, Clinicopathological characteristics, Prognosis, Survival

## Abstract

**Background:**

Epstein–Barr virus (EBV)-associated gastric cancer (EBVaGC) was a unique molecular subtype of gastric cancer (GC). However, the clinicopathological characteristics and prognostic role of EBV infection remains unclear. We aimed to evaluate the clinicopathological features of EBVaGC and its role on prognosis.

**Methods:**

EBV-encoded RNA (EBER) in situ hybridization method was used to evaluate the EBV status in GC. The serum tumor markers AFP, CEA, CA19-9 and CA125 of patients were detected before treatment. HER2 expression and microsatellite instability (MSI) status was evaluated according to established criteria. The relationship between EBV infection and clinicopathological factors as well as its role on prognosis were investigated.

**Results:**

420 patients were enrolled in the study and of 53 patients (12.62%) were identified as EBVaGC. EBVaGC was more common in males (*p* = 0.001) and related to early T stage (*p* = 0.045), early TNM stage (*p* = 0.001) and lower level of serum CEA (*p* = 0.039). No association could be found between EBV infection and HER2 expression, MSI status and other factors (*p* all > 0.05). Kaplan–Meier analysis revealed that both the overall survival and disease-free survival of EBVaGC patients were similar to that of EBV-negative GC (EBVnGC) patients (*p* = 0.309 and *p* = 0.264, respectively).

**Conclusion:**

EBVaGC was more common in males and in patients with the early T stage and TNM stage as well as patients with lower serum CEA level. Difference in overall survival and disease-free survival between EBVaGC and EBVnGC patients cannot be detected.

**Supplementary Information:**

The online version contains supplementary material available at 10.1186/s13027-023-00489-9.

## Introduction

Gastric cancer (GC) is the fifth most common tumor and the third most deadly cancer in the world. In 2018, there were 782,685 deaths because of GC globally [1]. The incidence of GC and mortality varies by region and is highly dependent on diet and Helicobacter pylori infection [2]. Although the survival rate of GC has increased because of the improved treatment, the prognosis of GC patients remains poor [3]. Therefore, significant biomarkers to predict the prognosis of GC patients and to achieve personalized treatment are need.

Epstein–Barr virus (EBV) is a gamma virus and was discovered in Burkitt lymphoma in 1964 [4]. EBV infection can be seen in infectious mononucleosis, nasopharyngeal carcinoma, GC, etc. Generally, EBV infection was seen in about 10% of GC patients [5]. According to The Cancer Genome Atlas (TCGA) research, GC can be classified into four molecular subtypes: chromosomal instable types, genomically stable, microsatellite instable and EBV-positive [6]. EBV-positive performance was defined as a potential EBV infection and monoclonal proliferation of cancer cells. In fact, EBV infection is usually determined by in situ hybridization of EBV-encoded RNA (EBER), which is a reliable method for detecting EBV infection [7]. A meta-analysis in 2016 showed that EBV infection was a risk factor for GC development [8]. Besides, patients with EBV infection presented a unique clinicopathological features, such as high infection rate in males, early stage, etc. [9]. However, the clinicopathological features and prognostic significance of EBV infection for GC patients remains controversial [10, 11].

Therefore, our study will mainly focus on that whether EBV infection can be a prognostic indicator for GC patients. EBER's RNA probe was employed to detect the tissues of GC. The clinicopathological characteristics together with its prognostic value were analyzed in this study.

## Materials and methods

### Materials

All subjects and experimental protocols have been approved by the Ethics Committee of the First Affiliated Hospital of Sun Yat-sen University in China. All patients have obtained written informed consent and the study was complied with the ethical guidelines in the Declaration of Helsinki.

Patients with gastric adenocarcinoma were included in our study. All of them underwent biopsy or surgery at the First Affiliated Hospital of Sun Yat-sen University, China, from January 2011 to September 2021. The patients included in the study should meet the following criteria: (1) Gastric adenocarcinoma confirmed by histology; (2) Underwent biopsy or gastrectomy; (3) Representative tumor masses, which can be fully evaluated for the presence of EBV. Exclusion criteria include: Chemotherapy, radiotherapy or chemoradiation before surgery.

All tumor samples were histologically classified by senior pathologists on the basis of the World Health Organization (WHO) classification system. The clinicopathological characteristics of patients were obtained by consulting medical archives. The classification and stage of gastric tumors were determined in line with the 8th edition of the International Union for Cancer Control/United States Gastric Cancer Joint Committee. Follow-up of the patient was done every 3 months in the first 2 years after surgery and every 6 months after. The deadline of follow-up was July 2020.

### Methods

#### EBV-encoded RNA (EBER) hybridization in situ

As previously reported in the literature [12], 4 mm sections embedded in paraffin were harvested which compiled with the manufacturer's instructions. EBV-encoded RNA oligonucleotide probes were employed to identify EBV in GC cells by ISH. EBV-positive nasopharyngeal carcinoma specimens confirmed previously were defined as positive controls and slides that were not treated with probes were used as negative controls. Samples with brown staining in tumor nuclei were considered positive. Divide samples into Epstein–Barr virus-associated GC (EBVaGC) and Epstein–Barr virus-negative (EBVnGC) according to the performance of EBER.

#### Detection of tumor markers

Tumor markers including serum carcinoembryonic antigen (CEA), alpha fetoprotein (AFP), carbohydrate antigen 125 (CA125) and carbohydrate antigen 19-9 (CA19-9) were measured in GC patients before treatment. On the basis of the clinical testing standard, the cut-off values for AFP, CEA, CA19-9 and CA125were 20 ug/mL, 5 ug U/mL, 35 U/mL and 35 U/mL, respectively. A measured value above the cut-offs was considered positive.

#### HER2 status assessment

HER2 status was evaluated by immunohistochemistry (IHC) and/or in situ hybridization (ISH) assays [13]. According to Hofmann's criteria in GC [14], samples with IHC 3+, or IHC 2+ and HER2 amplification by FISH were defined as HER2 positive and other samples were defined as HER2 negative.

#### Microsatellite instability status assessment

Patients were randomly selected for Microsatellite instability (MSI) status analysis by clinicians. The MSI status was determined by using IHC or polymerase chain reaction (PCR). As previously reported [15], expression of MMR proteins (PMS2, MSH6, MSH2 and MLH1) was evaluated by IHC. Microsatellite stable (MSS) was determined with expressions of all MMR proteins, and high MSI (MSI-H) was designated with at least one MMR protein in tumor cell nuclei were negative [16].

For PCR-based method, formalin-fixed paraffin-embedded tumor tissues confirmed by pathologist and matched whole blood samples were used for MSI assessment. Briefly, 5 consecutive paraffin-embedded tumor tissues with 10um thickness were harvested and DNA extraction was done with TIANquick FFPE DNA Kit (No. DP330, TIANGEN Company, China) and the matched blood DNA extraction was done with TIANamp Blood DNA Kit (No. DP318, TIANGEN Company, China). As reported in previous studies, PCR based amplification allows the detection of MSI by comparing and measuring the size of amplified DNA fragments from tumors and matched whole blood samples from the same patient by electrophoresis [17], and then PCR amplification was performed for six dinucleotide markers (BAT-25, BAT-26, NR-21, NR-24, NR-27, MONO-27 [18]. The PCR program comprised an initial 3 min at 95 °C followed by 30 cycles of 15 s at 94 °C and 45 s at 60 °C, which were followed by 30 min at 70 °C. MSI-H was designated as at least two markers with instability [16], whereas slides with instability at 1 microsatellite marker and those without instability were defined as MSI-low (MSI-L) and microsatellite stable (MSS) which both were defined as microsatellite stability (MSS) in our study.

#### Evaluation of clinicopathological characters of EBVaGC

Evaluation was performed following established morphological, histopathological, and immunophenotypic characteristics. Two independent gastrointestinal pathologists who have no idea about the clinical pathological data jointly examined the histological evaluation.

The histological type of gastric adenocarcinoma was classified according to WHO classification, and the GC was staged based on the 8th edition of the International Union for Cancer Control/United States Joint Committee on Gastric Cancer.

#### Statistical analysis

Cases lost due to follow-up and cases of death due to reasons other than GC were considered censored. Chi-square test or Fisher’s exact test was used to compare categorical variables and evaluate the association between EBV infection and clinicopathological parameters. Log-rank test was used to calculate the difference in survival between subgroups and Kaplan–Meier method was applied to calculate the probability of survival. Cox proportional hazards regression was employed to assess the effect of EBV infection on GC‐specific survival. All statistical analyses were two-sided tests and *p* < 0.05 were considered statistically significant. SPSS 13.0 (SPSS Inc. Chicago, IL) was employed for all the analysis.

## Results

### Characteristics of patients

**Fig. 1:**
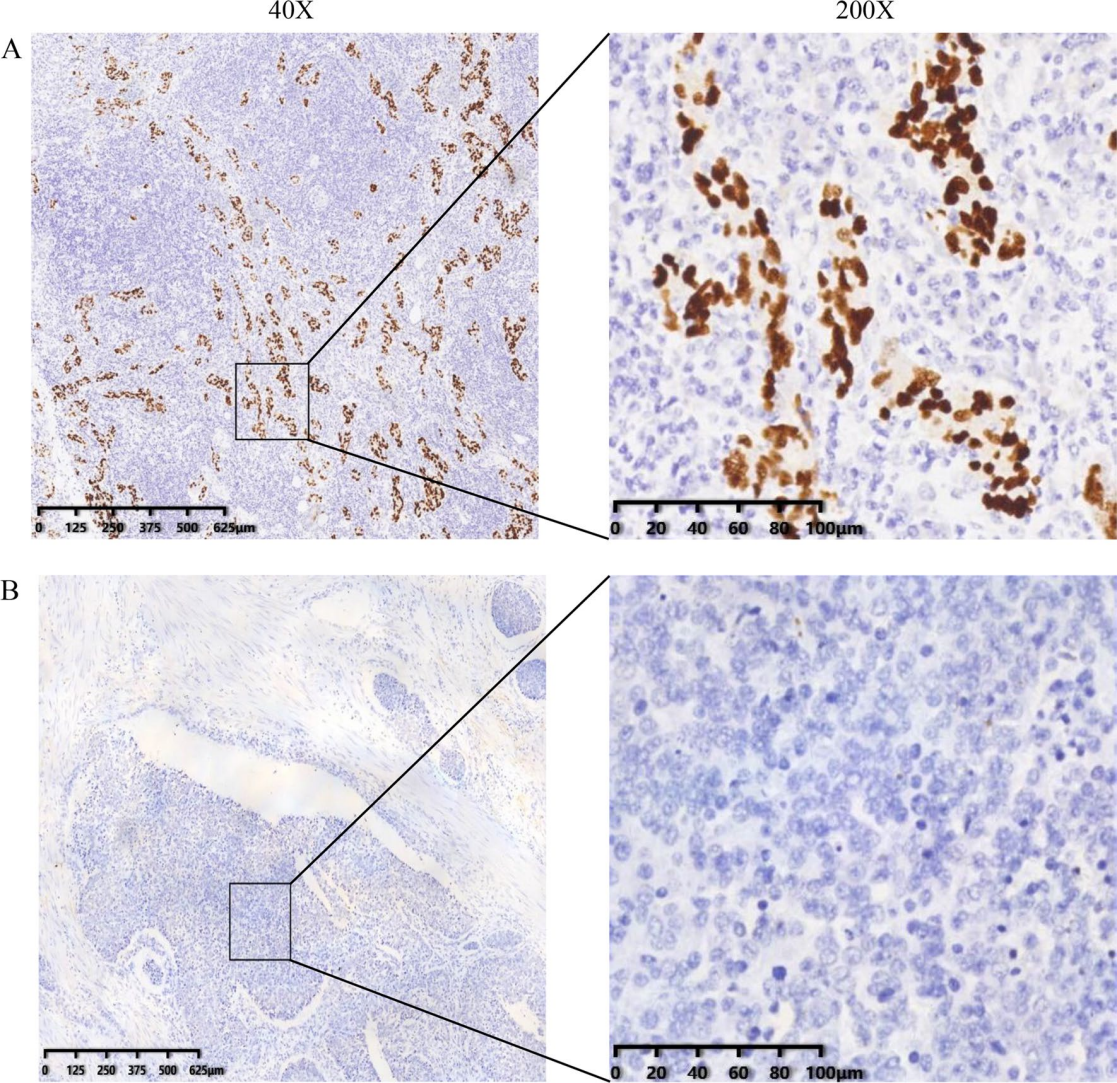
Representative images of EBV-encoded small RNAs (EBER) in situ hybridization which showed positive nuclei in the cancer cells in EBVaGC (40× and 200×). **A** Positive EBER staining in GC. **B** Negative EBER staining in GC

A total of 420 patients (284 males and 136 females) were included in this study. Of the 420 GC patients, 53 cases (12.62%) were EBVaGC patients, with 46 males and 7 females. The Clinicopathological features of all patients were shown in Table 1. All the EBVaGC showed EBER staining in 90% of tumor tissues (Fig. 1).

**Table 1:** Relationship between EBV infection and clinicopathologic characteristics

Characteristics	EBV infection		*p* value
	Positive	Negative	
*Sex*			0.001
Male	46	238	
Female	7	129	
*Age (years)*			0.421
≤ 60	31	193	
> 60	22	174	
*Location*			0.599
Proximal	14	109	
Distal	39	255	
*Tumor size*			0.799
≤ 5 cm	29	209	
> 5 cm	21	140	
*Differentiation*			0.146
Well	6	72	
Poor	47	295	
*WHO classification*			0.190
Well differentiated adenocarcinoma	6	72	
Poorly differentiated adenocarcinoma	46	271	
Mucinous adenocarcinoma	0	12	
Signet ring cell carcinoma	1	12	
*Tumor invasion depth*			0.045
T1–T2	18	79	
T3–T4	35	288	
*Lymph node metastasis*			0.099
N0	22	111	
N+	31	256	
*Distant metastasis*			0.124
M0	46	299	
M1	5	68	
*TNM stage*			0.001
I-II	30	121	
III-IV	22	245	
*MSI status*			0.275
MSS	10	41	
MSI-H	0	5	
*HER2 status*			0.928
Negative	9	64	
Positive	3	20	
*AFP*			0.322
Normal	39	262	
High	2	6	
*CA19-9*			0.247
Normal	36	215	
High	5	53	
*CA125*			1.000
Normal	26	242	
High	4	37	
*CEA*			0.039
Normal	47	221	
High	2	39	

*EBV* Epstein–Barr virus, *WHO* World Health Organization

EBVaGC was more common in males than women (*p* = 0.001) and related to early T stage (*p* = 0.045), early TNM stage (*p* = 0.001) as well as lower serum CEA level (*p* = 0.039). EBV infection status was not related to age (*p* = 0.421), tumor location (*p* = 0.599), histological type (*p* = 0.190), degree of differentiation (*p* = 0.146) and tumor size (*p* = 0.799) (Table 1). Moreover, there were no differences in HER2 expression, MSI status, serum AFP, CA19-9, CA125 levels between EBVaGC and EBVnGC patients (*p* all > 0.05).

### Correlation between EBV infection and overall survival (OS) of patients with gastric cancer

**Fig. 2:**
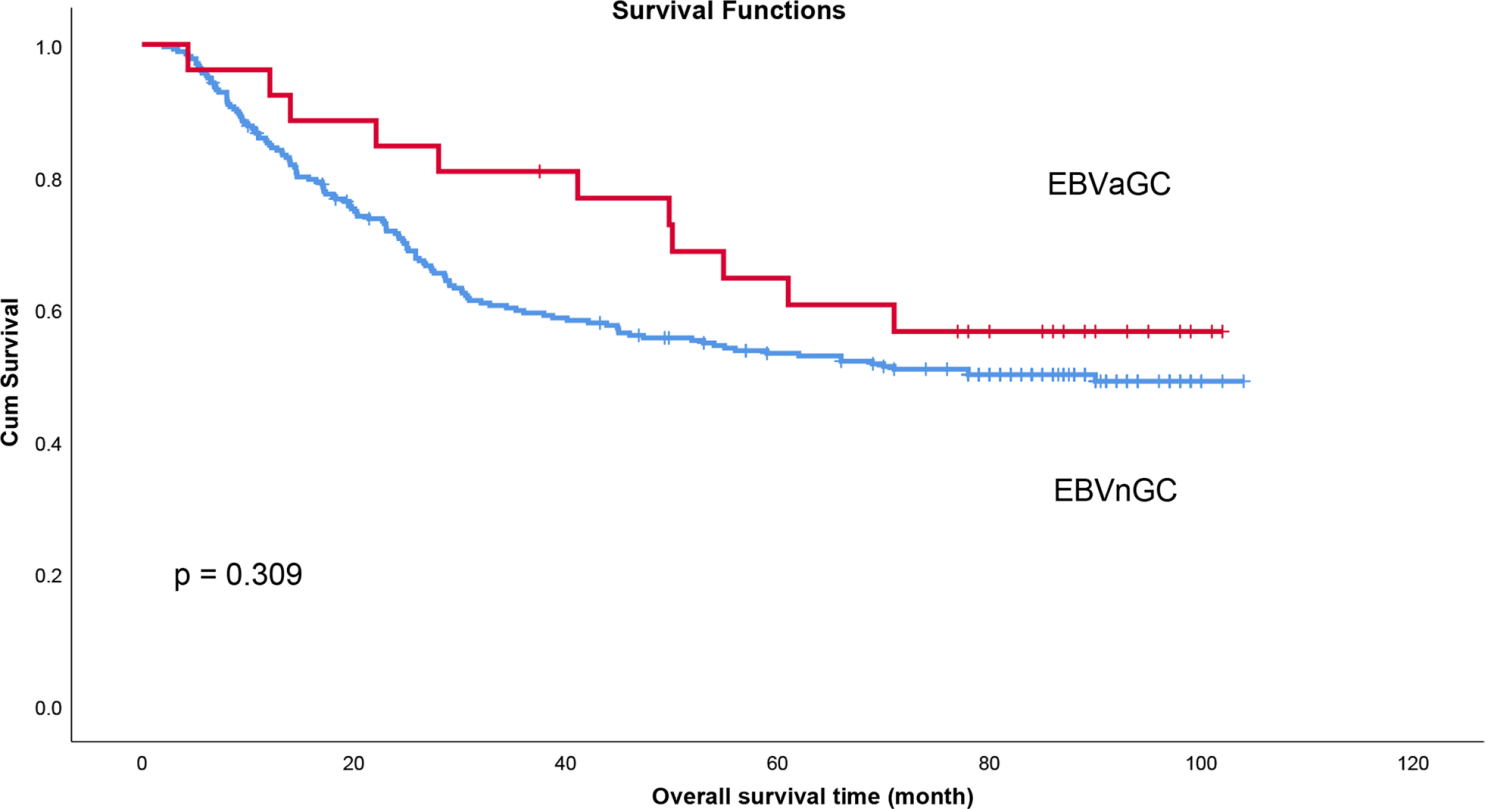
Comparison of overall survival of EBVaGC and EBVnGC patients

After removing the data of patients who died within a month, 303 patients who underwent gastrectomy from January 2011 to January 2013 were enrolled to analysis the relationship between overall survival (OS) and disease-free survival (DFS) and EBV infection. Baseline characteristics of patients were shown in Additional file 1. The median survival time for EBVaGC patients was 56.5 months, while the median survival time for EBV-negative patients was 59.0 months. However, survival analysis showed no significant difference in OS between EBVaGC and EBVnGC patients (*p* = 0.309) (Fig. 2). Multivariate analysis showed that TNM stage and tumor size were associated with the prognosis of GC patients (Additional file 2).

### Correlation between EBV infection and disease-free survival (DFS) of patients with gastric cancer

**Fig. 3:**
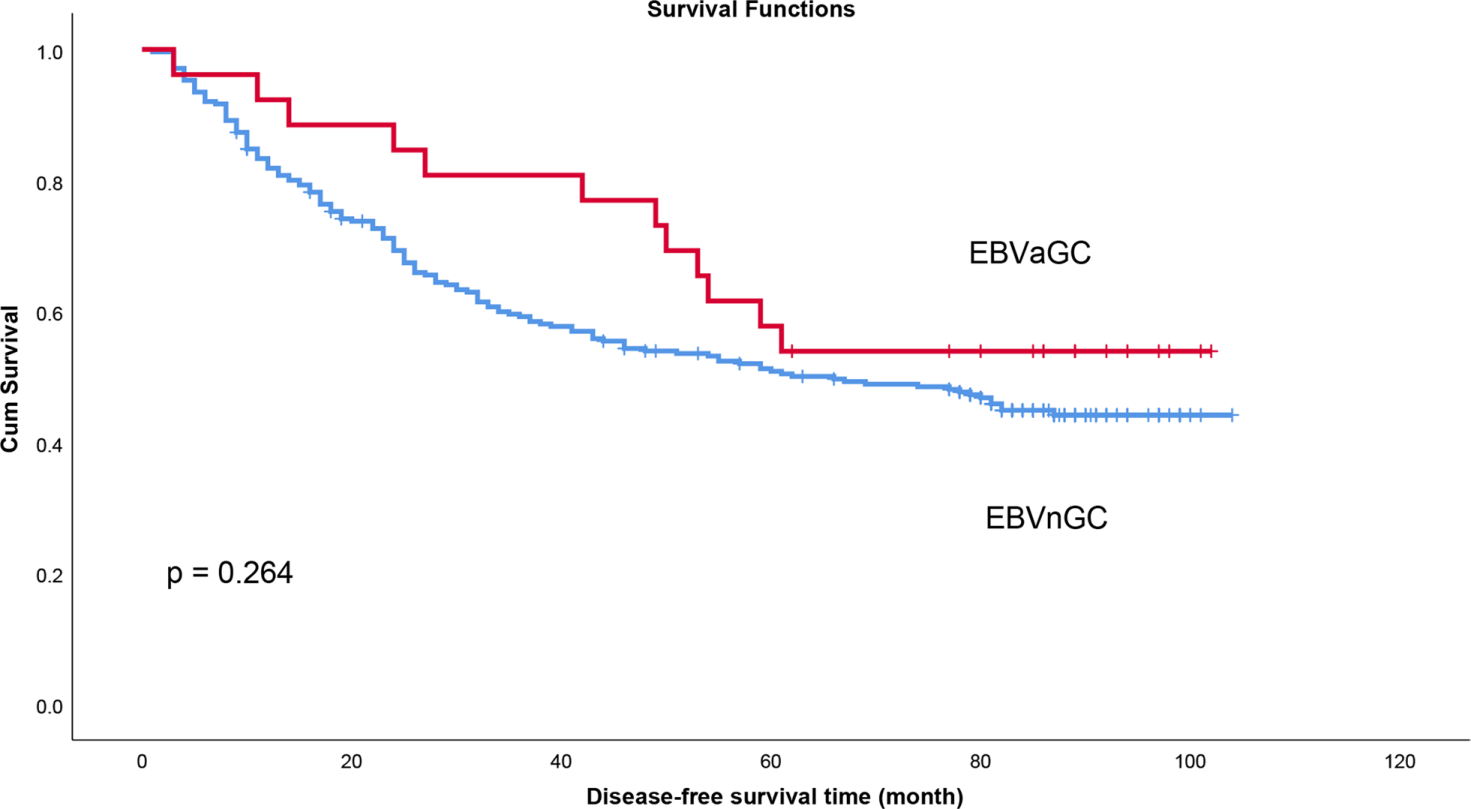
Comparison of disease-free survival of EBVaGC and EBVnGC patients

Survival analysis showed that DFS of EBVaGC and EBVnGC patients was not statistically different (*p* = 0.264) (Fig. 3). Univariate analyses and multivariate analyses illustrated that tumor size and TNM stage were all related to DFS, while EBV infection status was not related to DFS (Additional file 3).

## Discussion

In recent years, the correlation between EBV infection and the prognosis of GC remains to define. Our study showed no significant difference between the prognosis of EBVaGC and EBVnGC patients regardless of OS or DFS (*p* all > 0.05). Besides, EBV infection was found to be related to males, early T stage and TNM stage, and serum CEA level, suggesting that EBVaGC has unique clinicopathological features.

In our study, the overall survival of EBVaGC patients was shorter than that of EBVaGC patients (*p* = 0.306), which was consistent with some previous studies [19, 20]. However, some literatures reported better overall survival in EBVaGC patients [21, 22] while two studies reported that EBV infection was a worse prognostic indicator for patients with EBVaGC [23, 24]. A meta-analysis in 2015 showed that patients with EBV infection had a better prognosis, while accompanied by high heterogeneity, especially in different regions [10]. But one thing to mention is that this study included studies using detection methods of PCR and RNA sequence. However, the prognostic value of EBV infection is still debated as EBER has become the gold standard for detecting EBV [7]. Therefore, larger cases or a high-evidence meta-analysis are needed to unravel the prognostic value of EBV infection in GC patients.

In terms of DFS, no difference was detected between EBVaGC and EBVnGC patients in our study (*p* = 0.264). Two other recent papers also reported that EBV infection is not related to DFS [25, 26], consistent with our analysis. However, there is also some literature showing better DFS in EBVaGC patients [27, 28]. As GC is highly heterogeneous among individuals, it is necessary to study the gene expression differences between EBVaGC and EBVnGC patients, which may affect prognosis.

In our analysis, the EBV infection rate was 12.62%, close to the 10% infection rate reported in other literature [29, 30], so we can conclude that EBV infection in GC is common. Consistent with most studies [31, 32], we found that EBV-positive expression was more common in male patients (*p* = 0.001), and a meta-analysis showed the same conclusion [11], which may be related to smoking [33]. One study reported that cigarette smoke extracts could induce EBV reactivation in some EBV-positive cell lines [34], but it lacked high-level evidence. Though the reason for the gender difference remains to discover, the correlation between EBV infection and gender is positive.

As previous studies shown, EBV infection has been significantly correlated with some features, such as gender and tumor site [35]. A meta-analysis in 2020 show that is only correlated with gender and not with other clinical features [11], indicating that EBV infection is not associated with most clinicopathological features. Although many clinical features are controversial, EBVaGC has a unique mechanism, such as DNA methylation microRNAs, which affect carcinogenesis, tumor cell proliferation, apoptosis, etc. [36]. Moreover, EBVaGC has specific immune microenvironment, such as infiltrating immune cells and abundant PD-L1 expression [37–39]. These properties of EBVaGC will be the focus of future research.

As reported in previous studies, HER2 expression is usually detectable in EBVaGC [40], and we detected HER2 expression in 3 of 12 (25%) EBVaGC patients. According to Zang et al. [41] and Li et al. [42], HER2 expression is lower in EBVaGC cases than in EBVnGC cases. In accordance with the previous findings [43], our study also found no association between EBV infection and HER2 amplification (*p* = 0.928), possibly due to the low positive rate of EBV infection and HER2 expression, resulting in insufficient statistical power. The crosstalk between HER2 and EBV signaling pathways may affect gastric carcinogenesis and progression, such as the occurrence and enhancement of the epithelial-mesenchymal transition (EMT) event [44]. However, reports on the crosstalk mechanism are scarce, and more research is needed.

Besides, there was no cross case in EBV-positive and MSI-H molecular subgroups defined by TCGA in our study. EBVaGC and MSI-H GC contain similar epigenetic features, including high levels of DNA methylation in CpG islands, whereas CpG methylation is even more marked in the EBV-positive category than in the MSI class [45], and EBV-positive and MSI-H GCs are considered as mutually exclusive [16, 46]. A possible reason is that the tumor stemness reduce when MLH1 is silenced in EBV-positive GC cell lines [47]. Such exclusivity between EBV infection and MSI status is an interesting research topic, while they are already predictive markers of immunotherapy efficacy [48].

It is well known that the preoperative serum CEA levels and tumor CEA-positivity are positively correlated [49]. Our study found that EBV infection was associated with reduced serum CEA (*p* = 0.039), in line with a previous report that EBV infection is negatively correlated with CEA expression in tumor tissue [50]. The specific mechanism has not been studied. But there are existing studies showing that EBV interferes with TGF-β signal transduction [51], while TGF-β contributes to the stimulation of CEA transcription in GC cells [52]. Based on this, we infer that EBV could affect CEA expression. How EBV affects CEA production and secretion requires further study.

According to Seung Tae Kim [53], the overall remission rate of metastatic GC patients with EBV infection was 100% if they received anti-PD1 therapy. Previous studies have shown that PD-L1 expression is associated with EBV infection [54, 55] and the efficacy of immune checkpoint inhibitors can be predicted by detecting the status of EBV infection [53]. Therefore, EBV infection maybe not an indicator of prognosis if immunotherapy is not taken.

## Conclusions

Our study revealed the clinical and pathological characteristics of EBV-associated GC in south China. EBV infection was not a prognostic indicator for GC patients according to our analysis.

## Supplementary Information

**Additional file1**. Relationship between EBV infection and clinicopathologic characteristics.

**Additional file2**. Univariate and multivariate analysis of overall survival-related factors.

**Additional file3**. Analysis of univariate and multivariate factors affecting the disease-free survival of patients.

## Data Availability

The datasets used and/or analyzed during the current study are available from the corresponding author on reasonable request.
